# Early Effects of the Soluble Amyloid β_25-35_ Peptide in Rat Cortical Neurons: Modulation of Signal Transduction Mediated by Adenosine and Group I Metabotropic Glutamate Receptors

**DOI:** 10.3390/ijms22126577

**Published:** 2021-06-19

**Authors:** Carlos Alberto Castillo, Inmaculada Ballesteros-Yáñez, David Agustín León-Navarro, José Luis Albasanz, Mairena Martín

**Affiliations:** 1 Department of Nursing, Physiotherapy and Occupational Therapy, School of Physiotherapy and Nursing, University of Castilla-La Mancha, 45071 Toledo, Spain; carlosa.castillo@uclm.es; 2 Regional Center for Biomedical Research (CRIB), University of Castilla-La Mancha, 02071 Albacete, Spain; inmaculada.byanez@uclm.es (I.B.-Y.); davidagustin.leon@uclm.es (D.A.L.-N.); mairena.martin@uclm.es (M.M.); 3 Department of Inorganic, School of Medicine of Ciudad Real, Organic and Biochemistry, University of Castilla-La Mancha, 13071 Ciudad Real, Spain; 4 Department of Inorganic, Faculty of Chemical and Technological Sciences, Organic and Biochemistry, University of Castilla-La Mancha, 13071 Ciudad Real, Spain

**Keywords:** metabotropic glutamate receptors, adenosine receptors, Alzheimer’s disease, beta-amyloid

## Abstract

The amyloid β peptide (Aβ) is a central player in the neuropathology of Alzheimer’s disease (AD). The alteration of Aβ homeostasis may impact the fine-tuning of cell signaling from the very beginning of the disease, when amyloid plaque is not deposited yet. For this reason, primary culture of rat cortical neurons was exposed to Aβ_25-35_, a non-oligomerizable form of Aβ. Cell viability, metabotropic glutamate receptors (mGluR) and adenosine receptors (AR) expression and signalling were assessed. Aβ_25-35_ increased mGluR density and affinity, mainly due to a higher gene expression and protein presence of Group I mGluR (mGluR_1_ and mGluR_5_) in the membrane of cortical neurons. Intriguingly, the main effector of group I mGluR, the phospholipase C β_1_ isoform, was less responsive. Also, the inhibitory action of group II and group III mGluR on adenylate cyclase (AC) activity was unaltered or increased, respectively. Interestingly, pre-treatment of cortical neurons with an antagonist of group I mGluR reduced the Aβ_25-35_-induced cell death. Besides, Aβ_25-35_ increased the density of A_1_R and A_2A_R, along with an increase in their gene expression. However, while A_1_R-mediated AC inhibition was increased, the A_2A_R-mediated stimulation of AC remained unchanged. Therefore, one of the early events that takes place after Aβ_25-35_ exposure is the up-regulation of adenosine A_1_R, A_2A_R, and group I mGluR, and the different impacts on their corresponding signaling pathways. These results emphasize the importance of deciphering the early events and the possible involvement of metabotropic glutamate and adenosine receptors in AD physiopathology.

## 1. Introduction

Alzheimer’s disease (AD) is characterized by a progressive decline in cognitive functions, and the accumulation of amyloid β (Aβ) peptide (such as Aβ_1-42_) in senile plaques is one of the main hallmarks in AD [1]. It has been controversial the enigmatic roles of the different forms of Aβ peptide, derived from the metabolism of amyloid precursor protein (APP), in physiological and pathological conditions. It has been assumed for decades that APP has a prominent role in memory acquisition that could be mediated by Aβ peptide [2], but it has also been believed that Aβ_1-42_ peptide is synaptotoxic *per se* in the absence of plaque burdens [3]. In this sense, it has been known for a long time that the physiological production of soluble Aβ_1-40_ peptide is also a crucial factor for the maintenance of neurons viability [4]; even some authors propose that soluble purified monomers Aβ_1-42_ peptide may have a neuroprotective role [5,6]. It has been reported that Aβ monomers can protect primary cortical neurons against trophic deprivation and excitotoxicity by means of a mechanism involving the activation of the phosphatidyl-inositol-3-kinase (PI-3-K) pathway [5]. The protective effects of Aβ seems to be Aβ size-form specific, with the Aβ_1-42_ size form affording limited protection and the Aβ_25-35_ size form having very little protective effect [4]. Therefore, the question of the protective function of Aβ monomers seems to be underexplored [6,7]. While Aβ_1-42_ peptide is the Aβ form most prone to aggregation [8], Aβ_25-35_ form, a truncated form of Aβ_1-42_, represents the minimum functional domain of the Aβ peptide required for both neurotrophic and neurotoxic effects [9,10]. In this sense, Aβ_25-35_ has often been chosen as a model in structural and functional studies, which has contributed to the understanding of the effects of Aβ-mediated toxicity and has also facilitated the study of the modulation of its toxicity [11].

Furthermore, although Aβ accumulation into senile plaques is considered as an AD diagnostic tool, nowadays there is strong evidence that there are increased levels of soluble oligomer forms of Aβ_1-42_ peptide, the main cytotoxic form that is also responsible for electrophysiological, anatomical, and behavioural abnormalities reported in AD brains [12,13,14]. Despite all this knowledge supporting the “amyloid hypothesis”, researchers have not been able to develop an effective therapy based on the blockade of the increase of Aβ peptide. Therefore, some authors suggest that the role of Aβ in AD should be thoroughly challenged, and new ideas and explanations sought [15].

Glutamate is the main excitatory neurotransmitter in the mammalian nervous system, playing a key role in cognitive and motor functions. Glutamate-elicited responses are mainly mediated by specific receptors that have been classified into ionotropic and metabotropic glutamate receptors (mGluR). The latter belong to the G protein-coupled receptors (GPCR) superfamily and are the main modulators of glutamate action. Based on sequence homology, signal transduction mechanisms and agonist selectivity mGluR have been divided into three groups [16]. The mGluR_1_ and mGluR_5_ subtypes are included in Group I mGluR, whose canonical transduction pathway is the stimulation of the phospholipase C (PLC) system through G_q/11_ proteins, and they are the most studied receptors among the mGluR family. Different neurodegenerative diseases have been linked to changes in mGluR-mediated signalling [17]. Several years ago, the possible role of mGluR ligands, especially mGluR_5_, as neuroprotective agents in AD, was proposed [18]. Moreover, there is a growing body of evidence supporting the relationship between molecular changes in the glutamatergic system and the neurodegenerative processes triggered by Aβ in AD and other neurodegenerative diseases [19], even more since it was reported that Aβ interacts with mGluR_5_ [20]. In this sense, it has been described that Aβ_1-42_ oligomers promote the clustering and mobilization of mGluR_5_ on hippocampal cultured neurons, which suggests that mGluR_5_ might have a prominent role in the early-synaptic failure induced by Aβ [20]. Furthermore, the intracerebral administration of soluble Aβ_1-42_ induced a predominant loss of glutamatergic terminals, which correlates with the period where memory impairment appears with no affection of other terminals than glutamatergic [21] and that the long term depression (LTD) induced by soluble Aβ oligomers was mediated by NMDA or mGluR activity [22].

Adenosine is a widely distributed neuromodulator involved in a variety of physiological functions. Many of these actions are mediated by adenosine receptors (AR) that, like mGluR, are included in the GPCR superfamily. AR are subdivided into adenosine A_1_, A_2A_, A_2B_, and A_3_ receptors (A_1_R, A_2A_R, A_2B_R, and A_3_R, respectively), A_1_R and A_2A_R being the main mediators of adenosine action in the CNS [23]. The canonical transduction pathway of AR includes the interaction with the adenylyl cyclase (AC) system, A_1_R and A_3_R being able to inhibit AC through G_i/o_ proteins and A_2A_R and A_2B_R able to stimulate AC through G_s_ proteins [24]. Besides, AR alteration in human disease has been extensively studied. The levels and functionality of AR have been reported to be altered in several neurodegenerative diseases, including AD [25]. In this regard, while A_1_R activation may confer neuroprotection against neurotoxic stimuli, A_2A_R activation may contribute to neuronal injury through the enhancement of glutamate release [26].

Despite the solid evidence of the effect of soluble Aβ oligomers on neurons and/or other systems, little is known about the molecular changes into the glutamatergic and adenosinergic systems that takes place when the concentration of Aβ starts raising. We have previously reported the existence of cross-interaction between mGluR and AR [27], the modulation of AR [25] and mGluR [28] in the cerebral cortex from AD patients, and the modulation of these receptors in a mouse model of accelerated senescence [29,30]. Thus, the present work aimed to study the effect of the Aβ_25-35_ form in the functionality and expression of metabotropic glutamate receptors, mainly group I, and adenosine receptors, mainly A_1_R and A_2A_R, in rat primary cortical neurons.

## 2. Results

### 2.1. Characterization of Aβ-Induced Toxicity on Cortical Neurons

**Figure 1:**
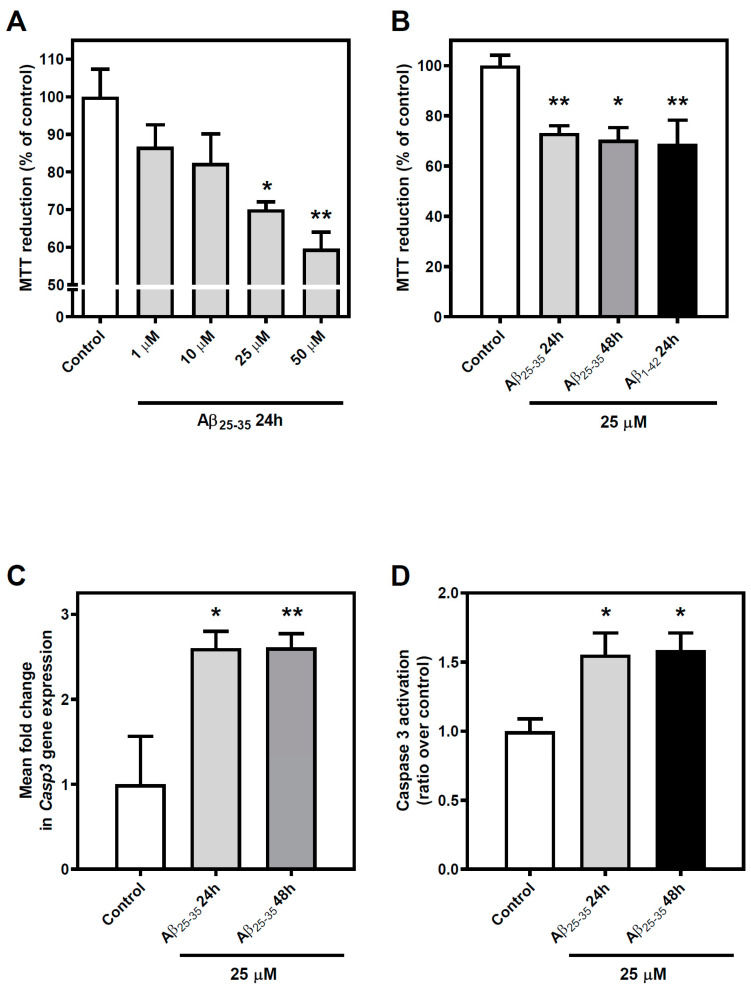
Effect of Aβ_25-35_ and Aβ_1-42_ exposure on cell viability of cortical neurons. (**A**) Cortical neurons were exposed to different concentrations of the soluble form of Aβ (Aβ_25–35_) (1–50 µM) for 24 h and MTT reduction was used as a measure of cell viability. Only high concentrations of Aβ_25–35_ (≥25 μM) significantly reduced cell viability under these conditions. (**B**) Cortical neurons were subjected to 25 μM Aβ_25–35_ for 24 and 48 h or to 25 μM Aβ_1–42_ for 24 h and cell viability was determined as described in Methods. No differences were reported when the exposition time was prolonged using Aβ_25-35_ or when Aβ_1–42_ was used instead Aβ_25-35_ in the same conditions. (**C**) Expression levels of Caspase 3 (*Casp3*) were determined under experimental conditions. Gene expression was incremented when cortical neurons were exposed to Aβ_25–35_. (**D**) Caspase 3 activity was increased by Aβ_25-35_ exposure. Mean ± SEM values obtained are represented; each point was measured in triplicate employing, at least four different cultures. * *p* < 0.05, ** *p* < 0.01 significantly different from control value using one-way ANOVA and Bonferroni’s post hoc test.

Cortical neurons were exposed to increasing concentrations (1, 10, 25, and 50 µM) of Aβ_25-35_ for 24 h or maintained under control conditions, and cell viability was measured using MTT reduction assay. A concentration-dependent decrease in cell viability was detected (Figure 1, panel A). Using the highest concentrations of Aβ_25-35_ (25 µM and 50 µM), a significant (*p* < 0.05 and *p* < 0.01, respectively) decrease in cell viability of almost 40% was observed. Nevertheless, the difference between those treatments was not statistically significant.

To check whether the effect of Aβ_25-35_ exposure was acute or not, we performed further experiments in which we tested the effect of exposure time in the reported decrease of cell viability induced by Aβ_25-35_. In these experiments, cortical neurons were exposed to 25 µM for 24 or 48 h, and again MTT was used to measure cell viability. We introduced Aβ_1-42_ (25 µM, 24 h) as a positive control to compare with the effects of the whole peptide form of Aβ (Figure 1, panel B). A decrease in cell viability was reported in all cases (at least *p* < 0.05), but no statistical differences were observed when the different conditions were compared to each other.

Finally, to complete the validation of this experimental model, we wanted to check that Aβ_25-35_ exposure maintained the Aβ_1-42_-mediated induction of apoptosis reported in the scientific literature. To this end, we studied the gene expression of the caspase 3 coding gene and its activity in cortical neurons exposed to Aβ_25-35_ or maintained under control conditions. On the one hand, exposure to 25 µM Aβ_25-35_ produces a significant increase in the gene expression of the caspase 3 coding gene (at least *p* < 0.05) at both 24 and 48 h of exposure (Figure 1, panel C). On the other hand, under these same experimental conditions, an increase (*p* < 0.05) in Aβ_25-35_-induced caspase 3 activity was observed (Figure 1, panel D).

These results confirm that Aβ_25-35_ exposure induces cell death in rat cortical neurons in a concentration-dependent manner and settle the conditions to establish our experimental model.

### 2.2. Aβ_25-35_ Exposure Effect on the Density and Affinity of Metabotropic Glutamate Receptors

To investigate the effect of Aβ_25-35_ exposure on the total number and affinity of mGluR, we performed a radioligand binding assay using L-[^3^H]glutamic acid as radioligand under conditions in which specific binding to other glutamate receptors was blocked, as described in Materials and Methods Section 4. Rat cortical neurons were exposed to Aβ_25-35_ (25 µM, 24 or 48 h) or maintained under control conditions and total mGluR levels were assessed (Table 1). Total mGluR density (B_max_) was markedly increased after Aβ_25-35_ exposure (61% over control value at 24 h, *p* < 0.01; 99% over control value at 48 h, *p* < 0.001) and mGluR affinity (1/K_D_) was significantly decreased (almost 50% of the control value, *p* < 0.01). These data suggest that Aβ_25-35_ exposure modulates mGluR, inducing an increase in total receptors density and in their affinity for glutamate.

### 2.3. Aβ_25-35_ Effect on Group I Metabotropic Glutamate Receptors’ Gene Expression and Protein Levels

**Figure 2:**
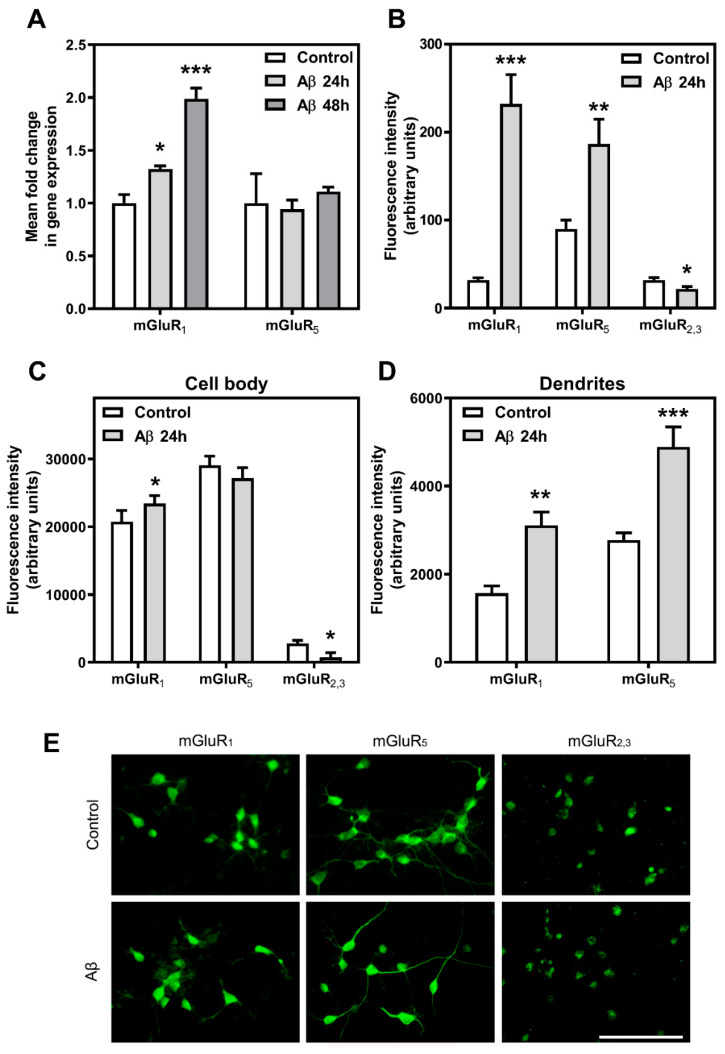
Aβ_25-35_ effect on mGluR gene expression and protein level. (**A**) Quantitative real time PCR assays after Aβ_25-35_ treatment were performed as described in Methods. Histograms represent mean ± SEM values obtained from, at least, three independent experiments performed in duplicate using different preparations. ** *p* < 0.01, *** *p* < 0.001 significantly different from control value using one-way ANOVA and Bonferroni’s post hoc test. The effect of Aβ_25-35_ treatment on mGluR_1_, mGluR_5_, and mGluR_2,3_ membrane protein level was assayed using image analysis of immunostaining in the whole-cell (**B**), the cell bodies (**C**), and the dendrites (**D**). Histograms represent mean ± SEM values obtained from, at least, three independent experiments performed in duplicate using different preparations. * *p* < 0.05, ** *p* < 0.01, *** *p* < 0.001 significantly different from untreated condition using two-tailed Student’s *t*-test. (**E**) Representative photomicrographs of mGluR_1_, mGluR_5_, and mGluR_2,3_ immunochemistry in control and Aβ_25-35-_treated cortical neurons. Bar represents 100 μm.

As the previous results indicate an important increase in total mGluR density and affinity, we tried to determine the specific modulation of Group I mGluR after Aβ_25-35_ exposure. To assess that possible modulation, we studied mGluR_1_ and mGluR_5_ gene expression and protein levels under control and treated conditions in cortical neurons. To analyse the possible modulation of genes coding for Group I mGluR proteins, namely GRM1 and GRM5, we performed real-time PCR with total RNA isolated from control and Aβ_25-35_ (25 µM, 24 or 48 h) treated cortical neurons. After Aβ_25-35_ exposure, cortical neurons showed an increase in mGluR_1_ gene expression (at least *p* < 0.05) but did not present any significant variation in mGluR_5_ gene expression when compared with control neurons (Figure 2, panel A).

Nevertheless, as gene expression levels do not always correlate with protein levels (30,36), we measured the protein levels of mGluR_1_, mGluR_5_, and mGluR_2,3_ at the membrane surface of cortical neurons exposed to Aβ_25-35_ (25 µM, 24 h) or maintained under control conditions by immunostaining in non-permeabilized cells employing extracellular specific antibodies (Figure 2, panel B). An increase in the density of mGluR_1_ and mGluR_5_ was detected at the membrane level after Aβ_25-35_ exposure, especially significant in the case of mGluR_1_ (mGluR_1_: more than 7-fold increase over control value, *p* < 0.001; mGluR_5_: twice over control value, *p* < 0.01). Interestingly, a further analysis by differentiating cell body and dendrite regions revealed that such increase was mainly observed in dendrites (Figure 2, panels C and D). Nevertheless, a decrease in the density of mGluR_2,3_ was observed at the membrane surface of cell bodies after Aβ_25-35_ exposure. Quantitation of each condition and representative micrographs are shown (Figure 2, panel E). Although immunofluorescence assays were performed with extracellular antibodies and without a permeabilization step, it could be possible that the fixation method employed here (10 min with 4% PFA at room temperature) affects the integrity of the plasma membrane. Therefore, the results of the immunofluorescence experiments should be viewed with caution. Together, these data indicate that Aβ_25-35_ exposure induces differential changes in mGluR proteins.

### 2.4. Modulation of PLC β_1_ Signalling Pathway after Aβ_25-35_ Exposure

As altered glutamatergic transmission has been suggested to have a pivotal role in many neurodegenerative diseases, we decided to further analyze the effect of Aβ_25-35_ exposure on the canonical transduction pathway mediated by Group I mGluR, the phospholipase C activation through G_q_ proteins.

**Figure 3:**
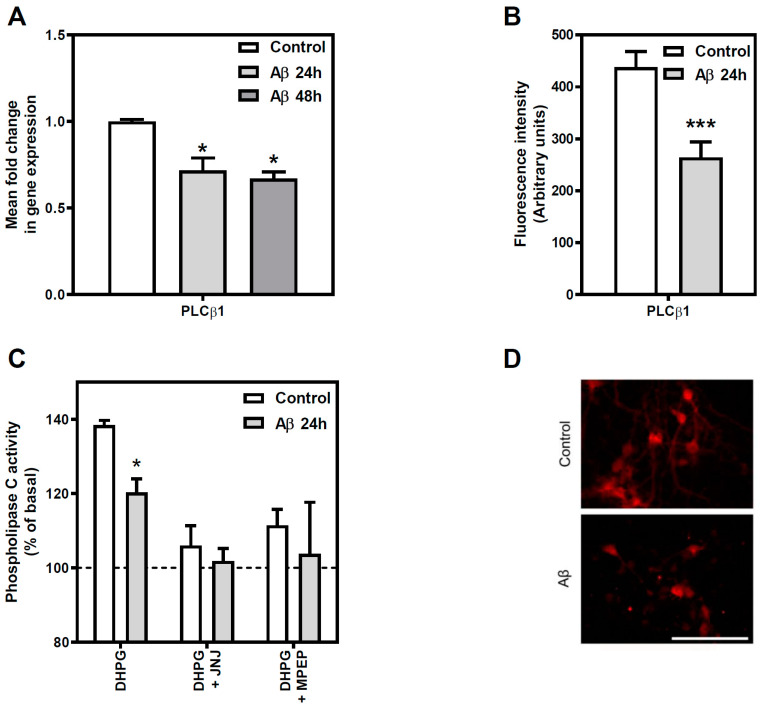
Effect of Aβ_25-35_ exposure on Group I mGluR-mediated PLC pathway in cortical neurons. (**A**) Effect of Aβ_25-35_ treatment on PLCβ_1_ gene expression was measured using qPCR. Rat cortical neurons were exposed to 25 μM Aβ_25-35_ for 24 h and 48 h or maintained under control conditions prior to RNA isolation. Quantitative real time PCR assays were performed as described in Methods. Data are means ± SEM of, at least, three independent RNA isolations and their quantitative PCR replicates. * *p* < 0.05 significantly different from control value using one-way ANOVA and Bonferroni’s post hoc test. (**B**) Effect of Aβ_25-35_ treatment on PLCβ_1_ protein level was measured using immunohistochemistry. Data are means ± SEM of, at least, three independent experiments performed in duplicate using different preparations. *** *p* < 0.001 significantly different from untreated condition using two tailed Student´s *t*-test. (**C**) Group I mGluR-mediated PLC activity was measured under control and treated conditions. Stimulation of phosphoinositide breakdown was measured in intact neurons exposed to 25 μM Aβ_25-35_ for 24 h or maintained under control conditions. Group I mGluR-mediated PLC activity was stimulated using 30 μM DHPG for 10 min in control and Aβ-treated cortical neurons. The specificity of the assay was tested using the antagonists JNJ 16259685 and MPEP at concentrations of 0.5 µM and 1 µM, respectively. Results are expressed as a percentage of the basal (unstimulated) activity in the absence of DHPG (basal values: control 26.81 ± 7.27; Aβ_25-35_ treated 17.61 ± 4.85 pmol/mg·min). Data are means ± SEM of, at least, three independent experiments performed in duplicate using different cultures. All sets of data were significantly different (at least *p* < 0.05) from their corresponding basal value. * *p* < 0.05, significantly different from control value using two tailed Student´s *t*-test. (**D**) Representative images of immunochemistry experiments presented in panel B are shown for control and 25 μM Aβ_25-35_ exposed cells for 24 h. Bar represents 100 μm.

We first tested whether gene expression of PLCβ_1_, the most highly expressed of PLCβ isoforms in the brain [31], was modulated in the presence of Aβ_25-35_. We determined PLCβ_1_ gene expression by real-time RT-PCR with total RNA isolated from control and Aβ_25-35_ (25 µM, 24 or 48 h) treated cortical neurons. We detected a significant decrease in the expression levels of this gene after Aβ_25-35_ exposure (28% and 33% decrease at 24 and 48 h over control value, respectively, *p* < 0.05) (Figure 3, panel A).

We then assessed whether PLCβ_1_ protein levels correlated with the observed decrease in its gene expression after Aβ_25-35_ exposure. For that purpose, we performed immunocytochemistry assays in cortical neurons fixed and permeabilized (Figure 3, panel B). The quantification of the images indicated that Aβ_25-35_ exposure reduced the total amount of PLCβ_1_ protein when compared to control cells (40% decrease over control value, *p* < 0.05). Representative micrographs of each condition quantified are shown in panel D of Figure 3.

To further analyze the Aβ_25-35_ effect on the main transduction pathway mediated by the group I mGluR, we performed enzymatic activity assays to determine the functionality of the whole system. Therefore, PLC activity was determined in basal and DHPG-stimulated conditions in cortical neurons exposed to Aβ_25-35_ (25 µM, 24 h) or maintained under control conditions (Figure 3, panel C). A significant decrease in PLC activity was observed in Aβ_25-35_ exposed cells when DHPG was used as an agonist (13% of decrease, *p* < 0.05), while no significant differences were observed when the activity was studied in basal conditions (basal activity: Control 26.8 ± 7.3 pmol/mg·min versus Aβ_25-35_ 17.6 ± 4.8 pmol/mg·min). Therefore, Aβ_25-35_ exposure reduced the functionality of the Group I mGluR/PLC signalling pathway, but no modulation of PLC basal activity was found. Besides, 0.5 µM JNJ 16259685 (a highly potent selective mGluR_1_ antagonist) or 1 µM MPEP (a potent mGluR_5_ antagonist), added 10 min before the addition of DHPG, were used to distinguish the specific involvement of mGluR_1_ and mGluR_5_ on PLC stimulation. These antagonists blocked DHPG-elicited PLC stimulation in control cells and there were no differences between control and Aβ_25-35_ treated cells (Figure 3, panel C).

### 2.5. Effect of Aβ_25-35_ Exposure on Metabotropic Glutamate Receptors/Adenylyl Cyclase Pathway

**Figure 4:**
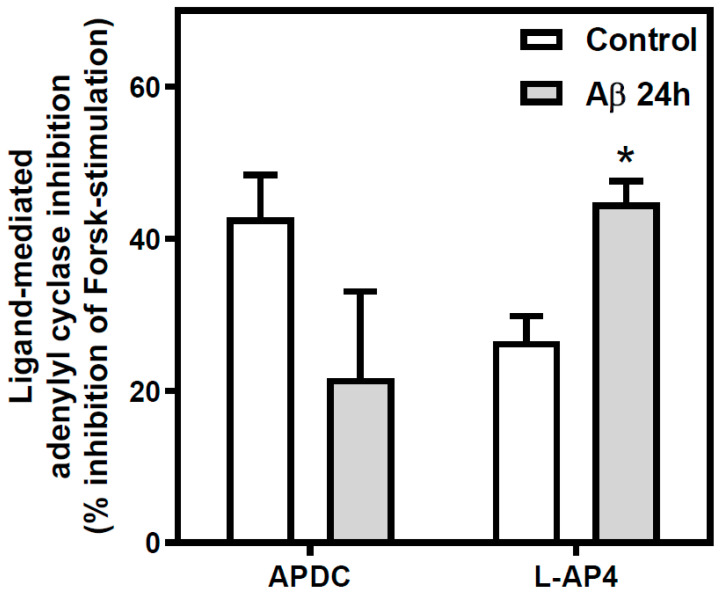
Effect of Aβ_25-35_ treatment on Group II and III mGluR-mediated adenylyl cyclase pathway in cortical neurons. Inhibition of AC was measured in intact neurons exposed to 25 μM Aβ_25-35_ for 24 h or maintained under control conditions. Control and treated cells were incubated in the presence of forskolin (5 µM) and in the absence or in the presence of 100 μM (2R,4R)-APDC or 100 μM L-AP4, specific group II and group III mGluR agonists, respectively. Aβ_25-35_ exposure increased Group III-mediated inhibition of the forskolin-stimulated AC activity in cortical neurons. Data are means ± SEM of, at least, three independent experiments performed in duplicate using different preparations. All data were significantly different (at least *p* < 0.05) from their respective forskolin-stimulated value. Basal values: control 2.91 ± 0.60; Aβ_25-35_ treated 2.33 ± 0.55 pmol/mg·min. * *p* < 0.05 significantly different from untreated condition using two tailed Student´s *t*-test.

To study whether proteins and gene expression variations detected in neurons were associated with alterations of their corresponding functionality, mGluR-mediated AC activity in control and Aβ_25-35_ (25 µM, 24 h) exposed neurons under basal and stimulated (5 µM forskolin, an activator of AC) conditions was determined. No differences were found in basal or forskolin-stimulated AC activity values between control and Aβ_25-35_-treated neurons (basal activity: control 2.91 ± 0.60 pmol/mg·min versus Aβ_25-35_ treated 2.33 ± 0.55 pmol/mg·min). Also, mGluR-mediated AC inhibition was performed using 100 µM APDC and 100 µM L-AP4, selective agonists for Group II and Group III mGluR, respectively, in the presence of 5 µM forskolin (Figure 4). While the ability to inhibit forskolin-mediated AC stimulation by Group II mGluR remained unaltered after Aβ_25-35_ exposure, Group III-mediated AC inhibition was incremented in cortical neurons exposed to Aβ_25-35_ (69% of increase over control value, *p* < 0.05).

### 2.6. Effect of Aβ_25-35_ Exposure on Adenosine A_1_ and A_2A_ Receptors

Adenosine A_1_R and A_2A_R levels were analyzed by radioligand binding assay using [^3^H]DPCPX or [^3^H]ZM241385, respectively, as radioligand in rat cortical neurons exposed to Aβ_25-35_ (25 µM, 24 or 48 h) or maintained under control conditions (Table 1). Total A_1_R density (B_max_) was markedly increased after Aβ_25-35_ exposure (265% over control value at 24 h, *p* < 0.01; 184% over control value at 48 h, *p* < 0.01), while A_1_R affinity (1/K_D_) was significantly reduced (between 4.7–6.5 less affinity than control value, *p* < 0.05). Total A_2A_R density (B_max_) was not altered after 24 h of Aβ_25-35_ exposure but it was significantly increased (80% over control value, *p* < 0.001) after 48 h of treatment. In contrast, no significant variation of A_2A_R affinity (1/K_D_) was observed. These data suggest that Aβ_25-35_ exposure induces an increase in total A_1_R density and a decrease in its affinity, while A_2A_R is also up-regulated, but later (48 h), without changes on receptor’s affinity.

### 2.7. Effect of Aβ_25-35_ Exposure on Adenosine Receptor Gene Expression

**Figure 5:**
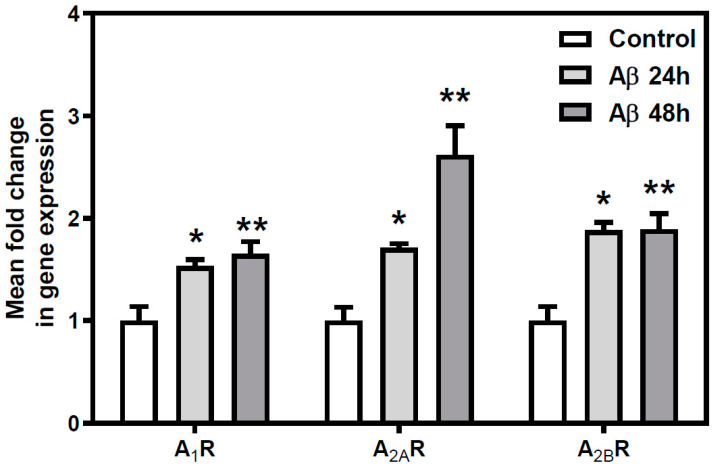
Aβ_25-35_ effect on AR gene expression. Quantitative real-time PCR assays after Aβ_25-35_ exposure were performed to measure the expression of A_1_R, A_2A_R, and A_2B_R genes. Histograms represent mean ± SEM values obtained from at least three independent experiments performed in duplicate using different preparations. * *p* < 0.05 ** *p* < 0.01 significantly different from control value using one-way ANOVA and Bonferroni’s post hoc test.

To determine whether variations observed in A_1_R and A_2A_R densities at the cell surface were due to modifications of gene expression, the expression of the three more common types of adenosine receptors, A_1_R, A_2A_R, and A_2B_R, were analyzed by real time PCR in neurons exposed to Aβ_25-35_ (25 µM, 24 or 48 h) or maintained under control conditions. In all tested conditions (Figure 5), A_1_R, A_2A_R, and A_2B_R gene expression were significantly increased (at least *p* < 0.05) by Aβ_25-35_ exposure. These results suggest that the increase in A_1_R and A_2A_R densities previously detected could be explained because of an increased gene expression.

### 2.8. Effect of Aβ_25-35_ Exposure on Adenosine Receptors-Mediated Adenylyl Cyclase Activity

**Figure 6:**
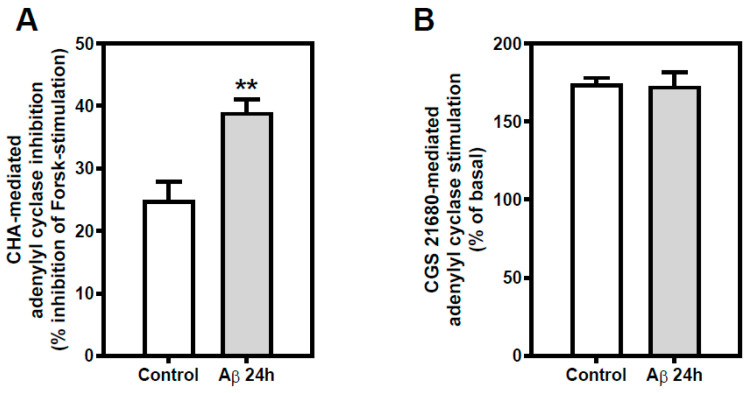
Effect of Aβ_25-35_ exposure AR-mediated adenylyl cyclase pathway in cortical neurons. (**A**) Inhibitory effect of 1 µM CHA, selective A_1_R agonist, on forskolin-stimulated cyclic AMP accumulation in intact cortical neurons was measured. Aβ_25-35_ exposure in cortical neurons increased A_1_R-mediated AC inhibition. (**B**) Stimulatory effect of 1 µM CGS 21680, selective A_2A_R agonist, was measured in intact neurons exposed to 25 μM Aβ_25-35_ for 24 h or maintained under control conditions. Histograms represent mean ± SEM values obtained from at least three independent experiments performed in duplicate using different preparations. * *p* < 0.05 ** *p* < 0.01 significantly different from control value using one-way ANOVA and Bonferroni’s post hoc test.

Adenosine A_1_R- and A_2A_R-mediated AC activity was measured in cortical neurons exposed to Aβ_25-35_ (25 µM, 24 h) or maintained under control conditions. Aβ_25-35_ exposure did not alter basal or forskolin-stimulated cyclic AMP levels, as commented above. A_1_R-mediated inhibition of AC activity (56% increase, *p* < 0.01), measured as the ability of 1 µM CHA to inhibit forskolin-mediated AC stimulation, was significantly increased (Figure 6, panel A). Nonetheless, Aβ_25-35_ exposure did not alter A_2A_R-mediated stimulation of AC activity, using 1 µM CGS 21680 as a selective A_2A_R ligand (Figure 6, panel B).

### 2.9. Effect of Aβ_25-35_ Exposure on the Expression of Genes Coding for CREB and CREM

**Figure 7:**
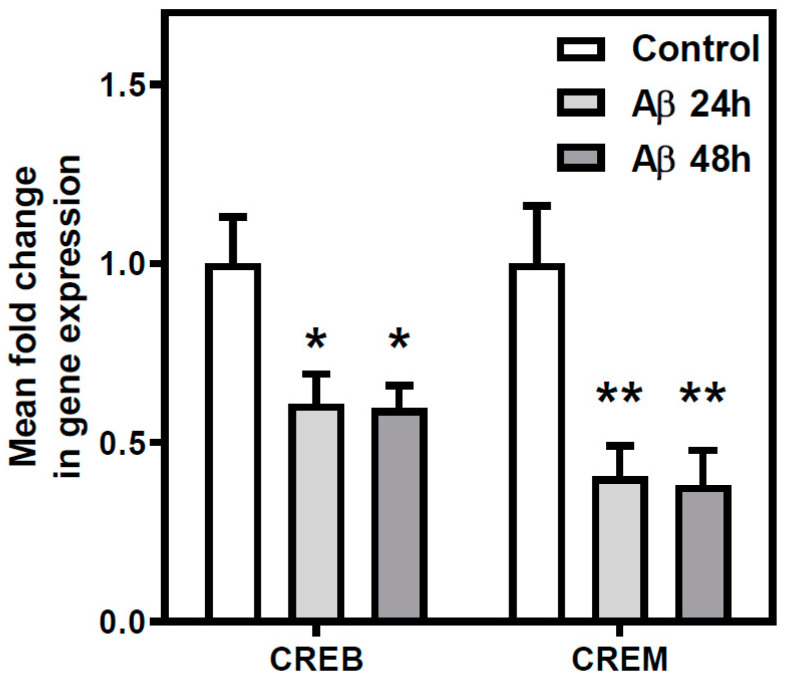
Aβ_25-35_ effect on the expression of cyclic AMP-dependent transcription factors. The expression of the cAMP-dependent transcription factors CREB and CREM was measured using qPCR. Rat cortical neurons were exposed to 25 μM Aβ_25-35_ for 24 h, 48 h, or maintained under control conditions prior to RNA isolation. Aβ_25-35_ exposure reduced the expression of CREB and CREM. Histograms represent mean ± SEM values obtained from three independent experiments performed in duplicate using different preparations. * *p* < 0.05 and ** *p* < 0.01 significantly different from control value using one-way ANOVA and Bonferroni’s post hoc test.

CREB and CREM transcriptional regulation factors gene expression was analyzed by real-time PCR in cortical neurons exposed to Aβ_25-35_ (25 µM, 24 or 48 h) or maintained under control conditions (Figure 7). Aβ_25-35_ exposure induced a decrease in the expression of genes encoding for CREB and CREM transcription factors in all tested conditions (at least *p* < 0.05). These results suggest that Aβ_25-35_ exposure could potentially affect molecular pathways regulated by CREB/CREM factors.

### 2.10. Effect of Group I Metabotropic Glutamate and Adenosine A_1_ and A_2A_ Receptors Ligands on Aβ_25-35_ Induced Toxicity

**Figure 8:**
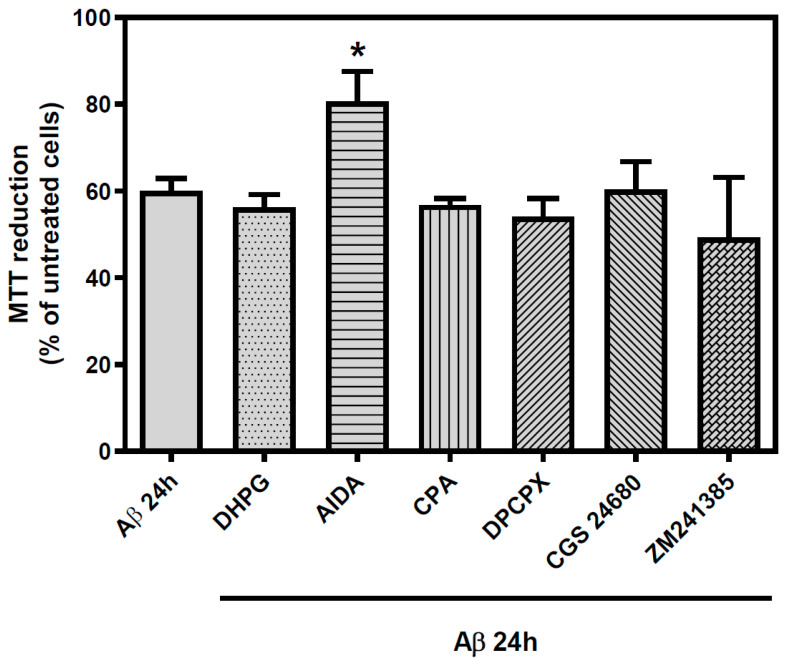
Effect of Group I metabotropic glutamate receptors and adenosine receptors selective ligands on the toxicity exhibited by Aβ_25-35_ exposure. Rat cortical neurons were subjected to selective Group I mGluR and AR ligands prior to 25 μM Aβ_25-35_ for 24 h. DHPG was employed as selective Group I mGluR agonist and AIDA as selective antagonist. CPA was used as selective agonist of A_1_R, DPCPX as A_1_R antagonist, CGS 21680 as A_2A_R agonist, and ZM241385 as A_2A_ antagonist. Each of the ligands were used at 100 µM and added 30 min before Aβ insult and maintained with amyloid peptide until the beginning of MTT quantification. Mean ± SEM values obtained are represented; each point was measured in triplicate employing, at least, three different cultures. * *p* < 0.05 significantly different from Aβ value using one-way ANOVA and Bonferroni’s post-hoc test.

Finally, to determine the possible neuroprotective role of these receptors, we tested whether the activation or blockade of Group I mGluR, A_1_R, or A_2A_R exerted any influence on cortical neurons viability (Figure 8). For that purpose, we used DHPG as selective Group I agonist and AIDA as selective antagonist; CPA and DPCPX as selective A_1_R agonist and antagonist, respectively; and CGS21680 and ZM241385 as selective A_2A_R agonist and antagonist, respectively. These drugs were added separately 30 min before the addition of Aβ_25-35_ (25 µM, 24 h). It was observed that Group I mGluR blockade with AIDA partially prevented Aβ_25-35_-induced toxicity (12% of viability increase in Aβ_25-35_ treated neurons, *p* < 0.05), while Group I activation or A_1_R or A_2A_R activation or blockade had no significant effect on Aβ_25-35_ induced toxicity. These data suggest that the blockade of Group I mGluR has a neuroprotective effect on cortical neurons exposed to Aβ_25-35_.

## 3. Discussion

Understanding the biological mechanisms underlying early alterations in AD is a key point to gain insight into AD etiopathogenesis and to define the appropriate time windows for AD treatment. The alteration of Aβ homeostasis, due to an imbalance between Aβ production and clearance, may impact the fine-tuning of synaptic vesicle cycling, neurotransmitter release, and cell signalling, thus altering synaptic homeostasis [32].

**Scheme 1:**
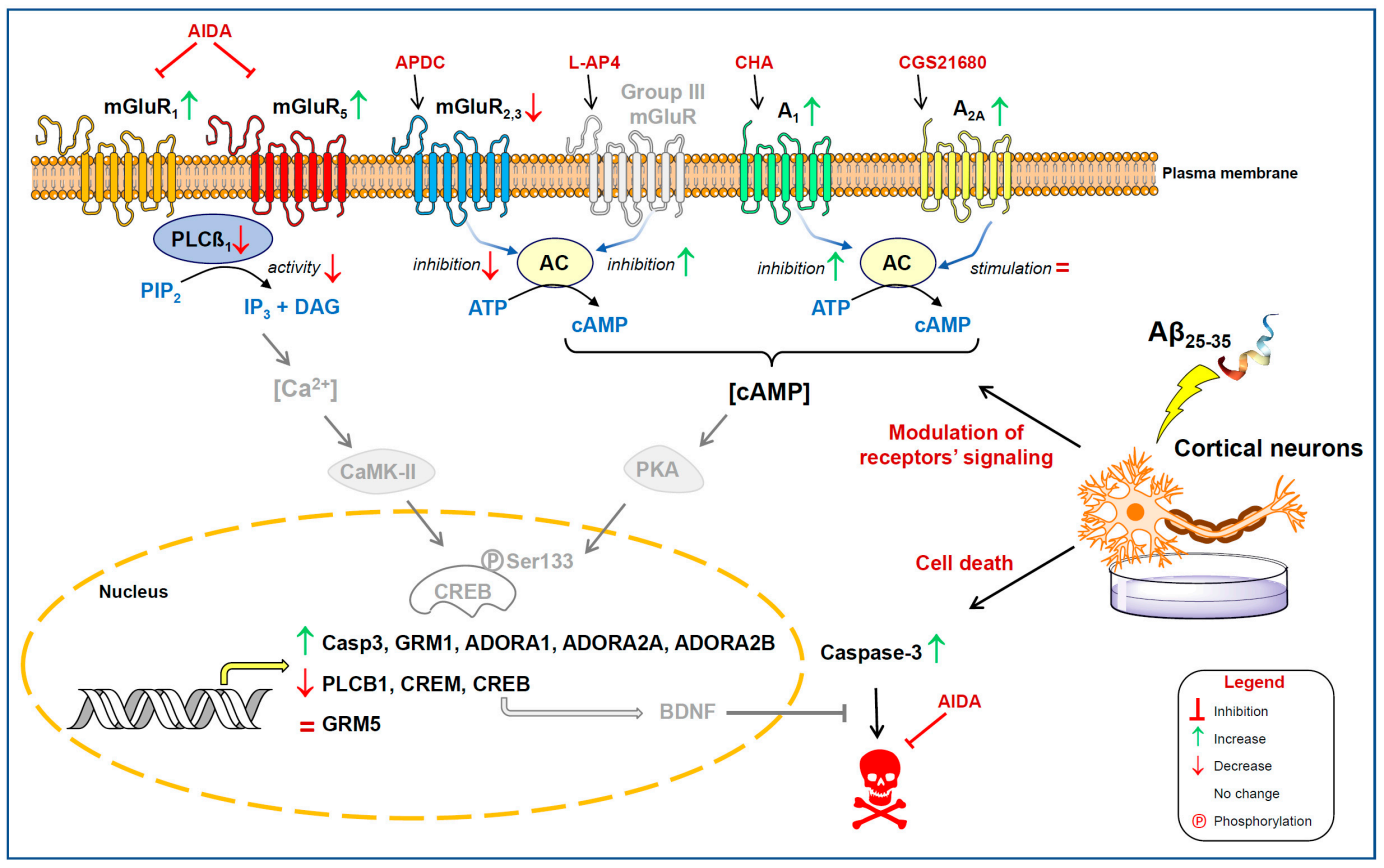
Early events after Aβ_25-35_ exposure. Density of metabotropic glutamate receptors (mGluR) and adenosine receptors (AR) are modulated at the plasma membrane (see Legend). Gene expression of indicated genes was also altered. Activation of phospholipase C (PLC) through mGluR was impaired. The ability of Group II (mGluR_2,3_) and Group III mGluR to inhibit adenylate cyclase (AC) activity was decreased and increased, respectively. The density of A_1_R and A_2A_R was increased together with a higher gene expression; however, while the ability of A_1_R to inhibit AC increased, the ability of A_2A_R to stimulate this system was unaffected by Aβ_25-35_ exposure. The gene expression of the transcription factors CREB and CREM was decreased. The cell death evoked by Aβ_25-35_ exposure was partially prevented by pre-incubation with a Group I mGluR antagonist (AIDA). Molecules or proteins not directly measured in the present study are shown in grey colour.

Our findings indicate that cortical neurons exposed to Aβ_25-35_, a peptide broadly used in in vitro and in vivo models of AD [33,34], undergo a significant change in the signalling pathways mediated by different mGluRs and ARs, and a reduction in the cell viability. In brief, (i) density of Group I mGluR was increased at the plasma membrane together with an increase in mGluR_1_ gene expression; however, the ability of these receptors to activate the PLC system resulted in being impaired. (ii) The ability of Group III mGluR to inhibit AC was increased. (iii) the density of A_1_R and A_2A_R was increased together with a higher gene expression; however, while the ability of A_1_R to inhibit AC was increased, the ability of A_2A_R to stimulate this system was unaffected by Aβ_25-35_ exposure. (iv) The gene expression of the transcription factors CREB and CREM was decreased. (v) Finally, the cell death evoked by Aβ_25-35_ exposure was partially prevented by pre-incubation with a Group I mGluR antagonist (Scheme 1).

Although a neuroprotective role of Aβ [5] that seems to be Aβ size-form specific [4] was reported, it has been assumed that Aβ_1-42_ soluble peptide could induce synaptic deficits before plaque deposition [35]. Our research group has reported the modulation of adenosine receptors, which is proposed as a new therapeutic target to manage AD cognitive deficits [36,37,38,39] in a model of ageing and age-associated diseases [29] at stages before the appearance of senile plaques [40]. Besides, mGluR are good candidates as molecular targets to reach neuroprotection in neurodegenerative diseases as they modulate (instead of mediate) excitatory synaptic transmission [17,18,41,42,43].

The ability of physiologically-released Aβ, particularly in oligomeric forms, to control neuronal excitability by inducing long term depression (LTD) through NMDA receptors activation has been known for a long time [44]. Furthermore, Aβ could induce this phenomenon by its ability to block neuronal glutamate uptake, leading to increased glutamate levels at the synaptic cleft; excessive NMDA receptors activation and consequently glutamate receptors internalization; lower increases in [Ca^2+^]_i_; and synaptic depression (reviewed in [3]). This hypothesis correlates with previous publications of our group. We described that in the frontal cortex of AD brains there is a down-regulation of total mGluR and a desensitization of the calcium signalling pathway mediated by PLC [28]. Also, we demonstrated that the mGluR expressed in cortical neurons exposed in vitro to glutamate (1 µM L-Glu for 24 h or 100 µM L-Glu for 2 h) suffer a classical response to agonist and are down-regulated at the time that excitotoxic cell death is induced [45]. Nevertheless, these previous data contrast with the results reported in the present work, focused on the very early effects of Aβ presence. Although the functional significance of this phenomena is unknown at present, the increase in Group I mGluR reported here could likely be part of a compensatory mechanism that manages to recover part of the lost synaptic transmission among neurons elicited by the toxic effect of Aβ. Differences between laboratory models (e.g., in vitro models) and human brains have been reported before and have been assigned to the fact that while the former serves as an example of a pre-clinical stage of the disease, the latter represents an advanced stage of the disease [46,47].

There is strong evidence that Group I mGluR develops a prominent role in the Aβ–neuron interaction, which could explain the up-regulation reported here. In this sense, soluble oligomers of Aβ induced the abnormal accumulation of mGluR_5_ in the synaptic cleft, and in reactive astrocytes [48], which produced synapse decline on hippocampal neurons [20]. Furthermore, although it was assumed that soluble Aβ oligomers disrupted synaptic plasticity by altering glutamate recycling at the synapse [49], recently it was proposed that those events should be secondary and that mGluR_5_ have a much more central role in mediating Aβ disrupting effects than was previously believed [50]. Our results correlate with those studies suggesting the interaction between mGluR_5_ and Aβ as the starter of a positive feedback mechanism, which induces a further increase in Aβ levels and, consequently, further neurodegeneration, a mechanism which can be prevented by genetic deletion of *GRM5*, which also would reduce Aβ burdens and soluble oligomers [51,52]. Therefore, the blockade of those receptors could also prevent this positive feedback. Interestingly, in the same animal model (APPswe/PS1ΔE9 mice), an age-dependent increase of mGluR_1_ in the cortex was reported, which is also related to the levels of Aβ peptide [53]. As we previously reported that mGluR_1_ levels decrease in the human brain frontal cortex with the progression of AD pathology [28], we suggest that mGluR_1_ levels could depend on the stage of disease and the cellular components (i.e., neurons, glia, etc.) mimicked by the experimental model.

In line with this, mGluR_5_ mRNA and protein level are upregulated in cultured astrocytes following 48 h of treatment with Aβ oligomers [54,55]. Moreover, it can be found a strong enrichment of mGluR_5_ on reactive astrocytes surrounding Aβ plaques and a rapid binding and clustering of Aβ oligomers over the astrocytic cell surface, which represents a diffusional trapping and clustering of mGluR_5_ within Aβ clusters, which in turn leads to an increased ATP release [48]. The mGluR_5_, with the participation of PrP^C^ as co-receptor [56,57], has been included among the candidates for receptors of Aβ oligomers [58].

Within the sequence of major pathogenic events leading to AD proposed by the amyloid cascade hypothesis, the very early increase in Aβ oligomers may directly injure the synapses and neurites of brain neurons, in addition to activating microglia and astrocytes [59]. Thus, a limitation of the present work could be the absence of other cell types also present in the whole brain compared to cortical neurons (e.g., absence of glial-neuronal interactions; however, it can be also helpful to avoid the presence of many other confusing factors in our experimental model that hinder our focus in the early events occurring in cortical neurons after Aβ peptide exposure. Therefore, these new data should be integrated with the effects described on other systems.

As the molecular mechanisms responsible for mGluR up-regulation are not understood, it is hard to explain the controversial effect of the specific antagonist (i.e., AIDA) on cell viability recovery. In fact, the precise role of Group I-specific drugs as neuroprotective agents or neurodegenerative facilitators has been discussed for a long time. Nowadays, the development of allosteric modulators seems to be a promising way to handle neurodegenerative disorders [60], although it is assumed that Group I mGluR antagonists are expected to behave as neuroprotective agents [18]. Different roles have been proposed for mGluR and their specific ligands depending on the system studied. Thus, mGluR_1_ could function as a dependence receptor, and while its overexpression is neurotoxic in rat cerebellar neurons, mouse cortical neurons or rat cortical astrocytes, the decrease of its expression is neuroprotective [61]. This neuroprotection by reduced receptor expression correlates with experiments presented here where the blockade of Group I receptors is partially neuroprotective. MGluR_1_ blockade might be a potential enhancer of GABA release [43,62], which could be responsible for the neuroprotective role reached by selective mGluR_1_ antagonists under various toxic conditions like ischemia [63,64] or in clinical environments for the treatment of anxiety, depression [65] or schizophrenia [42]. Moreover, selective blockade of mGluR_5_ is neuroprotective in vitro against Aβ toxicity [66] or in vivo against MPTP effects [67,68], with promising clinical perspectives also in other CNS-related diseases (reviewed in [69]).

The role of Aβ peptide in calcium signalling is also controversial. Differences between the experimental models that used non-oligomerizable forms of Aβ and the ones that employ Aβ burdens have been reported. In the first case, soluble Aβ peptide impairs PKC signalling [70] and decreases the NMDA receptor-mediated calcium signalling [71]. In the second case, some neurons in the vicinity of Aβ plaques experimented with an increase in intracellular calcium [72,73], and those neurons were directly related to the learning impairment observed in animal models. We have not measured calcium levels in the present study. However, we detected a decreased mGluR/PLC signalling after Aβ_25-35_ exposure. Furthermore, we previously reported this abnormal mGluR/PLC signalling in Diffuse Lewy body disease, a neurodegenerative disease with some AD-related hallmarks [74]. Those apparent controversial results concerning calcium levels must be further studied taking into account the dual role of glutamate signalling in physiology and pathology [75] and the possible role that modulation of G_q/11_ proteins under different conditions may have in Group I mGluR-mediated calcium signalling via PLC [76].

In the present work, the A_1_R density at the plasma membrane was modulated by Aβ_25-35_ before (24 h) than A_2A_R (48 h) and the grade of change was higher for A_1_R. This suggests a prominent role for A_1_R in the regulation of A_2A_R. Similarly, A_1_R activation was the starter of a coordinated program of re-adaptation of both A_1_R- and A_2A_R-mediated pathways found during hypoxia in rat C6 glioma cells, which were resistant to cell death elicited by hypoxic insult. Interestingly, CREB and CREM transcription factors were also decreased [77]. A_1_R was found to be significantly increased in human neuroblastoma SH-SY5Y cells treated with Aβ_25-35_ and in the brain tissue of 5xFAD mice when Aβ_25-35_ was directly injected into the lateral ventricles [78]. Furthermore, these results are consistent with the significant increase of both A_1_R and A_2A_R previously detected in the frontal cortex of AD patients from stages I to VI of Braak [25], although the different expression and activity of adenosine receptors seem to be brain region-specific (for a review see [37,38]).

The efficacy of caffeine (AR antagonist) against AD and AD-related cognitive impairment was reviewed, focusing on the proposed protective mechanisms of action [79]. There is evidence that caffeine and A_2A_R antagonists afford protection against Aβ-induced amnesia in vivo [80] and prevent the neuronal cell death caused by exposure of rat cultured cerebellar granule neurons to 25 µM Aβ_25-35_ for 48 h [81]. However, a neuroprotective role for A_1_R antagonism cannot be ruled out. In line with this, the blockade of both A_1_R and A_2A_R was responsible for beneficial effects of caffeine in human neuroblastoma SH-SY5Y cells exposed to Aβ_25-35_ alone [82] or combined with AlCl_3_ [83], probably because A_1_R blockade might potentiate A_2A_R-mediated protection by promoting the recovery of Ca^2+^ homeostasis [83]. Neuroprotection against some neurodegenerative disorders can be induced via promoting CREB/BDNF signalling pathway [84]. To further complicate this scenario, it was suggested that Aβ monomers (not oligomers), by activating the IGF-IR-stimulated PI3-K/AKT pathway, induce the activation of CREB in neurons and sustain BDNF transcription and release [85]. The upregulation of A_1_R reported here could be responsible for the lower levels of CREB expression and the downregulation of the anti-apoptotic CREB-driven gene expression. However, as DPCPX (i.e., A_1_R antagonism) was ineffective against cortical neuron cell death, a possible role for A_1_R and its enhanced inhibition of cAMP generation could be counteracting the increase of A_2A_R and its role in stimulating cAMP levels. Preclinical studies, clinical trials, and reviews suggest that increasing cAMP with phosphodiesterase inhibitors is disease-modifying in AD [86].

The present study provides strong evidence that one of the early events that takes place when cortical neurons are exposed to Aβ_25-35_, is the up-regulation of Group I mGluR and the desensitization of their main transduction system; the activation of PLC. Interestingly, the pre-treatment with an antagonist of these receptors is enough to reduce cellular damage. Besides, adenosine receptors (mainly A_1_R) are also early increased by Aβ_25-35_ exposure and their signalling pathway modulated in accordance, leading to enhanced inhibition of the formation of cAMP and, perhaps, a lower expression of transcriptional factors like CREB and CREM.

The strategies against AD based on lowering Aβ levels or reducing Aβ production in human trials are not as effective as first expected [14,15], so there is a chance to improve those strategies by focusing on the early events occurring in AD brains rather than the neurodegeneration caused by the amyloid plaques [87,88], and targeting adenosine receptors and/or Group I mGluR. Even the latter has the potential to mediate, at least in part, the influence of Aβ peptide on neurons. Of interest, apart from the interplay between Aβ and AR [89,90,91] or mGluR [41,57,92], the widely reported cross-talk between adenosine and metabotropic glutamate receptors could be helpful in the therapeutic intervention based on these receptors [27]. Moreover, different to other GPCRs that may have a neuroprotective role, Group I mGluR does not desensitize and down-regulate quickly, as does A_1_R [39], so there is a window of time in which these receptors could be considered potential targets for AD. Furthermore, the clear up-regulation of Group I mGluR on Aβ-treated neurons shows it is a promising diagnostic tool to detect the beginning of AD (promising imaging studies have been carried out in rodents [93]), and we would not have to wait a long time (usually 10–20 years) to observe the classical neurodegenerative phenotypes in AD patients, where neurodegeneration is too high to improve any kind of neuroprotective therapy [87]. The role of mGluRs in synaptic plasticity and their modulation as a possible strategy for AD intervention deserves further exploration, as recently reviewed [41]. Overall, this study increases our knowledge of the very early events that take place in neurons after soluble Aβ exposure and could provide new ways of targeting AD. Future research should study the molecular mechanisms underlying the processes described here that will help us to better understand the nature of these early interactions of Aβ with cortical neurons.

## 4. Materials and Methods

### 4.1. Materials

L-[3,4-^3^H]-glutamic acid (L-[^3^H]Glu, 52 Ci/mmol) and Inositol-1,4,5-trisphosphate (Inositol-1-[^3^H](N) ([^3^H]IP_3_, 24.1 Ci/mmol) were purchased from Perkin Elmer (Boston, MA, USA). Cyclopentyl-1,3-dypropylxanthine,8-[dipropy-2,3-^3^H(N)] ([^3^H]DPCPX, 120 Ci/mmol) was purchased from Amersham (Madrid, Spain). L-glutamic acid (L-Glu), (S)-3,5-dihydroxyphenylglycine (DHPG), (RS)-1-Aminoindan-1,5-dicarboxylic acid (AIDA), (3,4-Dihydro-2H-pyrano[2,3-b]quinolin-7-yl)-(cis-4-methoxycyclohexyl)-methanone (JNJ 16259685), 2-Methyl-6-(phenylethynyl)pyridine hydrochloride (MPEP), (2R,4R)-4-Aminopyrrolidine-2,4-dicarboxylate (APDC), L-(+)-2-Amino-4-phosphonobutyric acid (L-AP4), ([2-^3^H](4-(2-[7-amino-2-(2-fury1) [1,2,4] triazolo [2,3-a] [1,3,5] triazin-5-ylamino]ethyl)phenol) ([^3^H]ZM241385, 27.4 Ci/mmol), 8-Cyclopentyl-1,3-dipropylxanthine (DPCPX), 2-[p-(2-carboxyethyl) phenylamino]-50-N-ethylcarboxyamide adenosine (CGS21680) and 4-(2-[7-Amino-2-(2-furyl) [1,2,4] triazolo [2,3-a] [1,3,5] triazin-5-ylamino] ethyl)phenol (ZM241385) were from Tocris (Bristol, UK). (RS)-α-Amino-3-hydroxy-5-methylisoxazole-4-propionic acid (AMPA), 2-Carboxy-3-carboxymethyl-4-isopropenylpyrrolidine (kainic acid), (R)-2-(Methylamino)succinic acid (NMDA), threo-2-Amino-3-hydroxysuccinic acid (β-OH-Asp), N^6^-cyclopentyladenosine (CPA), N^6^-cyclohexyladenosine (CHA), calf intestine adenosine deaminase (ADA; EC 3.5.4.4), forskolin, cytosine β-D-arabinofuranoside (AraC), Aβ_25-35_, Aβ_1-42_, and 3-[4,5-dimetylthiazol-2-yl]-2,5-dipheniltetrazolium bromide (MTT) were obtained from Sigma (Madrid, Spain). Liquid scintillation solutions were purchased from Perkin Elmer (Boston, MA, EEUU). Liquid scintillation solutions were purchased from PerkinElmer (Boston, MA, USA). Chemicals, culture media, and culture plates used to obtain and maintain cellular cultures were acquired from Gibco BRL (Barcelona, Spain) unless otherwise stated. All other products were of analytical grade. All the drugs employed in this study were prepared, stored at −80 °C, and thawed only once. In all cases, appropriate controls were carried out to avoid the solvent effect.

### 4.2. Primary Culture of Cortical Neurons

Primary cortical neuronal cultures were prepared using Wistar rat foetuses on embryonic day 18 using a protocol described previously [94] with minor modifications [95]. Briefly, foetuses were isolated and placed in PBS supplemented with 6 mM glucose and 1% BSA. Cortical hemispheres were dissected under sterile conditions and incubated with papain (30 U/mL; Sigma, Madrid, Spain) for 5 min at 37 °C. Tissue was mechanically dissociated with a glass Pasteur pipette, then DNase was added, and dissociated cells were filtered through a 70 µm cell strainer (BD Falcon, Madrid, Spain). Cells were collected by centrifugation (300× *g* for 6 min) and resuspended in MEM (Minimum Essential Medium) supplemented with 2.2 g/L NaHCO_3_, 10 mL/L Glutamax I, 2.6 g/L HEPES, 10 mL/L Antibiotic-Antimycotic mix, and 10% decomplemented horse serum at a density of 4·10^5^ cells/mL. Neurons were plated at a density of 2.6·10^5^ cells/well on poly-D-lysine-coated 24-well plates (binding, PCR and enzymatic assays), on 12-mm-diameter poly-D-lysine-coated coverslips (immunochemistry), or at a density of 8·10^4^ cells/well on poly-D-lysine-coated 96-weel plates (viability assays). The following day medium was replaced with Neurobasal medium supplemented with B27. At 2 days in vitro (DIV), 5 µM AraC was added to inhibit glial growth, which was less than 5%. Cultures were kept at 37 °C in a 5% CO_2_ atmosphere. During the following weeks, half of the medium was changed once a week. All experiments were performed on neurons at 14–18 DIV. All experiments followed the European Community Council Directives (86/609/EEC) about animal experimentation, those of the Experimentation Animal Committee of Castilla-La Mancha University, and all efforts were performed to minimize the number of animals and their suffering.

### 4.3. Drug Treatments and Evaluation of Cell Death

Aβ_25-35_ and Aβ_1-42_ were water-soluble peptides, however, while Aβ_25-35_, the functional domain of Aβ required for both neurotrophic and neurotoxic effects, was directly added to neuronal cultures after its solubilisation, Aβ_1-42_ was solved in water and maintained for 24 h at 37 °C before its addition to promote its aggregation. To test the effect of mGluR ligands on cell death induced by Aβ in neuronal cultures, these drugs were added 30 min before the addition of Aβ_25-35_ and maintained during Aβ exposure. Unless otherwise indicated, all chemicals were dissolved in water.

Cell viability was determined on 96-well plates using an in vitro toxicology assay kit based on a mitochondrial-dependent reaction that transforms 3-[4,5-dimetylthiazol-2-yl]-2,5-diphenyltetrazolium bromide (MTT) into formazan crystals. Briefly, at the end of treatments, cells were incubated in culture medium with MTT solution (5 mg/mL) at 37 °C for 3 h. After incubation, MTT solubilization solution (10% Triton X-100 plus 0.1 M HCl in isopropanol anhydrous) was added into the wells to dissolve formazan crystals and the absorbance of each well was measured at 570 nm and 690 nm, according to the manufacturer’s instructions.

### 4.4. Caspase 3 Activity Assay

A commercial kit (Molecular Probes, Barcelona, Spain) was used to determine Caspase 3 activity following the manufacturer’s instructions. Briefly, cortical neurons growth in 24-well plates were exposed to Aβ_25-35_ or maintained under control conditions. Cells were lysed and lysates centrifuged to clear cell debris. Then, 50 µL of test buffer containing Z-DEVD-Rhodamine 110, DTT, EDTA, PIPES, and CHAPS was added to the supernatants obtained in the proportions indicated by the manufacturer. After 30 min of incubation at room temperature, the absorbance of each sample was measured on a plate reader (excitation/emission 496/520 nm).

### 4.5. Quantification of Metabotropic Glutamate and Adenosine Receptors in Intact Cells by Radioligand Binding Assay

Metabotropic glutamate receptors were determined using the radioligand L-[^3^H]glutamate, as described previously [45]. Radioligand binding assays using intact cells were performed in 24-well plates. Briefly, cells were washed with serum-free DMEM buffered with 20 mM HEPES pH 7.4 and incubated for 60 min at 37 °C in a final volume of 250 µL, in the presence of 100 μM AMPA, 100 μM NMDA, and 100 μM kainate, to block glutamate binding to these receptor types; 10 μM of the glutamate uptake inhibitor D,L-threo-β-hydroxyaspartic acid; and increasing concentrations of L-[^3^H] glutamate (200 nM to 1.2 µM). Non-labeled L-Glutamate in a concentration of 10^5^ times higher than the radioligand was used to determine non-specific binding. After incubation, cells were washed with 500 µL of ice-cold buffer and disrupted with 0.2% SDS. Well contents were then transferred to vials and a scintillation liquid mixture was added to measure radioactivity. At least two wells from each plate were reserved for protein concentration measurement.

Concerning adenosine receptor assessment by radioligand binding, [^3^H]DPCPX and [^3^H]ZM241385 were employed as selective A_1_R and A_2A_R radioligands, as described previously [25], to determine A_1_R and A_2A_R density, respectively. Briefly, primary cortical neurons growth in 24-well plates were washed with serum-free DMEM and pre-incubated with 2 U/mL ADA at 37 °C for 30 min to remove endogenous adenosine. After incubation, the radioligands [^3^H]DPCPX (1–20 nM) or [^3^H] ZM241385 (1–20 nM) were added in the absence or the presence of CPA in a concentration 10^4^ times higher than the radioligand, to obtain non-specific binding to A_1_R, or 5 mM theophylline, to obtain non-specific binding to A_2A_R. A specific adenosine uptake inhibitor (1 µM dipyridamole) was added to the reaction mixture to block adenosine receptor ligands’ binding to adenosine transporters. After incubation at 25 °C for 2 h in a final volume of 250 µL, cells were washed with 500 µL of ice-cold buffer and disrupted with 0.2% SDS. Well contents were then transferred to vials and a scintillation liquid mixture was added to measure radioactivity. Two wells from each plate were reserved for protein concentration measurement.

### 4.6. Extracellular Targeting of mGluR_1_, mGluR_5_ and mGluR_2,3_ by Immunochemistry

The quantitative analysis of the levels of Group I mGluR and mGluR_2,3_ at the plasma membrane was performed with extracellular targeted polyclonal antibodies (mGluR_1_, mGluR_5_ and mGluR_2,3_, Alomone Labs, Jerusalem, Israel), as previously described [96,97,98] with minor modifications. Briefly, after 2 weeks in culture, the culture medium was removed and cells washed with Locke Buffer (LB, pH 7.4). Cells were then fixed with 4% paraformaldehyde for 10 min at room temperature and washed with three 10-min intervals with LB.

Nonspecific staining was suppressed by blocking with 3% normal goat serum in LB for 60 min. Thereafter, the cells were incubated overnight at 4 °C in the same blocking buffer containing rabbit anti-mGluR_1_ (1:200) or anti-mGluR_5_ (1:200). Thereafter, cells were washed with LB, mounted following standard procedures using ProLong Gold as an antifade reagent, and stored in cold and dark conditions.

### 4.7. PLCβ_1_ Immunocytochemistry

For PLCβ_1_ immunostaining, the protocol described above was followed with the introduction of modified steps. Before the nonspecific staining blockade with 3% normal goat serum, cells were pre-treated with Triton 0.25% in LB for 10 min to induce membrane permeabilization. Cells were incubated overnight in blocking buffer with mouse anti-PLCβ_1_ monoclonal antibody (1:500) from Millipore (Bedford, MA, USA). The following day, after three LB washes (10 min each), cortical neurons were exposed to Cy3-conjugated goat anti-mouse IgG (1:600). In all immunochemical procedures, internal controls were carried out where primary antibodies were not added to the blocking buffer.

### 4.8. Microscopy Imaging

To evaluate the degree of fluorescence intensity in cortical neurons, fluorescence was measured to estimate the protein expression in the different experimental conditions analyzed. Images (448.6 µm × 335.08 µm) obtained with a digital camera (Leica DFC350FX R2), attached to a Leica DMI6000B (Leica Microsystems, Wetzlar, Germany) fluorescent microscope, were used for quantification (20x HCX PL FLUOTAR 0.4 dry objective). For whole-cell immunostaining, six ROIs were randomly distributed in five different images of each condition (N = 30 per experimental condition; 1324.16 µm^2^ each ROI). Fluorescence intensity was estimated by averaging mean grey values. These ROIs have enough size to include one to four cells. Data were normalized to the total number of cells in the analyzed fields. The mean fluorescence background, calculated in the unstained regions between neurons, was subtracted from the fluorescence intensity of each data point. For cell body and dendrite localized immunostaining, 10 ROIs were analysed in the same five images of each condition used for whole-cell immunostaining analysis. ImageJ 1.53 e (NIH, Bethesda, Maryland, USA) was used to measure cell fluorescence after selection of cell and dendrite portions using selection tools (freeform and rectangle, respectively). In “set measurements” display, the area-integrated intensity and mean grey value were used to calculate Correct Total Cell Fluorescence (CTCF) as CTCF = Integrated Density— (area of selected cell × Mean fluorescence of background readings). This method is based on QBI Advanced Microscopy Facility of The University of Queensland. All the samples were processed together. Acquisition parameters were adjusted to prevent fluorescence from saturating the image in any condition, and these parameters were maintained with the subsequent images.

### 4.9. Determination of Phospholipase C Activity

This method was previously reported [45] and is divided into two well-defined steps:

IP_3_ accumulation: culture medium was discharged, and cells were washed twice with DMEM containing 20 mM HEPES, pH 7.4. After 20 min at 37 °C, different ligands were added to cells for 10 min at 37 °C to analyse their effect on PLC activity in a final reaction volume of 300 µL. The enzymatic reaction was stopped and cells lysed by adding 50 µL 2.8 M perchloric acid and placing plates on ice for 30 min. The medium was neutralized with 70 µL 1 M Tris-HCl, 2 M KOH, and 60 mM EGTA, transferred to Eppendorf tubes and centrifuged at 12,000× *g* for 10 min. The supernatant was then collected to determine the IP_3_ level.

IP_3_ level determination: IP_3_ levels in cells were determined as previously reported by Palmer [99] and modified by Gerwins [100]. Incubation of [^3^H]IP_3_ (0.2 pmol; 3000–5000 cpm) in assay buffer (100 mM Tris-HCl, 4 mM EDTA, 4 mM EGTA, 4 mg/mL BSA, pH 9.0), binding protein (500–600 µg), and 25–50 µL of supernatant obtained from IP_3_ accumulation assay was carried out. Samples were incubated at 4 °C for 60 min and centrifuged at 12,000× *g* for 10 min. Pellets were suspended in 100 µL 0.2% SDS and transferred to scintillation vials to measure radioactivity. A standard curve was made with known [^3^H]IP_3_ concentrations (0.25–10 µM). Nonspecific binding was determined in the presence of 10 µM unlabeled IP_3_. Binding protein was obtained from bovine suprarenal capsules according to Palmer’s protocol [99].

### 4.10. Determination of Adenylyl Cyclase Activity

AC activity was determined as previously reported by Murphy [101] with minor modifications [77]. Briefly, control or treated cells were pre-incubated in serum-free DMEM buffered with 20 mM HEPES, pH 7.4, supplemented with 100 µM Ro 20-1724 and 2 U/mL ADA, for 10 min at 37 °C. AC activity was stimulated using specific ligands for 15 min at 37 °C in a final volume of 250 µL. The reaction was stopped by adding 500 µL 0.1 M HCl in absolute ethanol. Once centrifuged at 12,000× *g* for 10 min, supernatants were evaporated in speed-vac and resuspended in 150 µL assay buffer (50 mM Tris-HCl, 4 mM EDTA). Fifty microliters of the sample were used to determine cAMP accumulation using protein kinase A as a cAMP binding protein and [^3^H]cAMP as radioligand. A cAMP standard curve (0–16 pmol) was prepared in the same buffer. Incubation was stopped by rapid filtration through Whatman GF/B filters.

### 4.11. Preparation of Total RNA and cDNA

Total RNA was extracted using an ABI 6100 Nucleic Acid PrepStation according to the manufacturer’s protocol (Applied Biosystems Inc., Foster City, CA, USA). Total RNA from cells was isolated and stored at −80 °C. The quality of isolated RNA was estimated by the ratio A_260_/A_280_ and was in the range of 1.9–2.1. RNA integrity was evaluated by agarose gel electrophoresis according to MIQE guidelines [102] using the “bleach gel” method [103]. RNA concentrations were determined from the A_260_. One microgram of total RNA was reverse transcribed using Applied Biosystems High-Capacity cDNA Archive Kit.

### 4.12. Quantitative Real-Time RT-PCR Analysis

To assess relative gene expression in neurons, quantitative real-time RT-PCR analysis was performed with an Applied Biosystems Prism 7500 Fast Sequence Detection System using TaqMan^®^ universal PCR master mix according to the manufacturer’s specifications. The TaqMan probes and primers for GRM1 (assay ID: Rn00566625_m1), GRM5 (assay ID: Rn00566628_m1), ADORA1 (assay ID:Rn00567668_m1), ADORA2A (assay ID: Rn00583935_m1), ADORA2B (assay ID:Rn00567697_m1), PI-PLCβ1 (assay ID: Rn01514511_m1), CREB1 (assay ID: Rn00578826_m1), CREM (assay ID: Rn00565271_m1), Caspase3 (assay ID: Rn00563902_m1), and ACTβ (Rn00667869_m1) were validated assay-on-demand gene expression products. ACTβ gene was used as an endogenous control. A non-fluorescent quencher and the minor groove binder were linked at the 3′ end of the probe as quenchers. The thermal cycler conditions were as follows: hold for 20″ at 95 °C, followed by two-step PCR for 40 cycles of 95 °C for 3″ followed by 60 °C for 30″. Levels of RNA expression were determined using the 7500 Fast System SDS software version 1.3.1 (Applied Biosystems) according to the 2^−ΔΔCt^ method. Briefly, expression results of a gene were normalized to internal control β-actin and relatively to a calibrator, consisting of the mean expression level of the control gene as follows: 2^−ΔΔCt^ = 2^-((Ct target gene − Ct β-actin)sample − (Ct target gene − Ct β-actin)calibrator)^. The results from three independent repeat assays, performed in different plates each using different cDNA’s from the cultures analyzed, were averaged to produce a single mean quantity value for each mRNA.

### 4.13. Protein Determination

Protein concentration was measured by the method of Lowry, using bovine serum albumin as standard.

### 4.14. Statistics and Data Analysis

Statistical analysis was performed using non paired two-tailed Student’s *t*-test or one-way ANOVA, followed by Bonferroni’s multiple comparisons test, as appropriate. Differences between mean values were considered statistically significant at *p* < 0.05. Nonlinear regression curve fit (one site binding hyperbola) was performed for binding data using GraphPad Prism 8 program for Windows (GraphPad Software, San Diego, CA, USA).

## 5. Conclusions

We show strong evidence that one of the early events that takes place after Aβ_25-35_ exposure is the up-regulation of adenosine A_1_R, A_2A_R, and group I mGluR, and the different impacts on their corresponding signaling pathways. These results emphasize the importance of deciphering the early events and the possible involvement of metabotropic glutamate and adenosine receptors in AD physiopathology.

## Figures and Tables

**Table 1:** Aβ_25-35_ exposure effect on receptors density and affinity in cortical neurons.

	**Control**	**Aβ_25-35_ 24 h**	**Aβ_25-35_ 48 h**
**Total mGluR**			
B_max_ (pmol/mg prot)	590 ± 31	952 ± 9 **	1178 ± 93 ***
K_D_ (µM)	3.069 ± 0.062	1.607 ± 0.423 **	1.522 ± 0.163 **
**A_1_R**			
B_max_ (fmol/mg prot)	149 ± 6	545 ± 101 **	423 ± 66 *
K_D_ (nM)	1.16 ± 0.16	7.51 ± 2.35 *	5.51 ± 1.33 *
**A_2A_R**			
B_max_ (fmol/mg prot)	656 ± 51	557 ± 56	1178 ± 43 ***
K_D_ (nM)	13.26 ± 0.26	10.33 ± 1.22	13.96 ± 4.69

Saturation binding curves of L-[^3^H]Glutamate (200 nM to 1.2 µM), [^3^H]DPCPX (1 to 20 µM), and [^3^H]ZM241385 (1 to 20 µM) were performed in intact cortical neurons under control and treated conditions to measure total mGluR, A_1_R, and A_2A_R, respectively. Table shows the influence of Aβ_25-35_ treatment (25 μM, 24 h and 48 h) on total receptor density (B_max_) and affinity (1/K_D_). Data are mean ± SEM values obtained from five independent experiments performed in duplicate using different cultures. * *p* < 0.05, ** *p* < 0.01, and *** *p* < 0.001 significantly different from corresponding control value using one-way ANOVA and Bonferroni’s post hoc test.

## Data Availability

Data is contained within the article.
